# Design of a glutamine substrate tag enabling protein labelling mediated by *Bacillus subtilis* transglutaminase

**DOI:** 10.1371/journal.pone.0197956

**Published:** 2018-05-30

**Authors:** Samuel K. Oteng-Pabi, Christopher M. Clouthier, Jeffrey W. Keillor

**Affiliations:** Department of Chemistry and Biomolecular Sciences, Centre for Catalysis and Research Innovation, University of Ottawa, 30 Marie-Curie, Ottawa, Ontario, Canada; University of Canterbury, NEW ZEALAND

## Abstract

Transglutaminases (TGases) are enzymes that catalyse protein cross-linking through a transamidation reaction between the side chain of a glutamine residue on one protein and the side chain of a lysine residue on another. Generally, TGases show low substrate specificity with respect to their amine substrate, such that a wide variety of primary amines can participate in the modification of specific glutamine residue. Although a number of different TGases have been used to mediate these bioconjugation reactions, the TGase from *Bacillus subtilis* (bTG) may be particularly suited to this application. It is smaller than most TGases, can be expressed in a soluble active form, and lacks the calcium dependence of its mammalian counterparts. However, little is known regarding this enzyme and its glutamine substrate specificity, limiting the scope of its application. In this work, we designed a FRET-based ligation assay to monitor the bTG-mediated conjugation of the fluorescent proteins Clover and mRuby2. This assay allowed us to screen a library of random heptapeptide glutamine sequences for their reactivity with recombinant bTG in bacterial cells, using fluorescence assisted cell sorting. From this library, several reactive sequences were identified and kinetically characterized, with the most reactive sequence (YAHQAHY) having a k_cat_/K_M_ value of 19 ± 3 μM^-1^ min^-1^. This sequence was then genetically appended onto a test protein as a reactive ‘Q-tag’ and fluorescently labelled with dansyl-cadaverine, in the first demonstration of protein labelling mediated by bTG.

## Introduction

**Fig 1:**
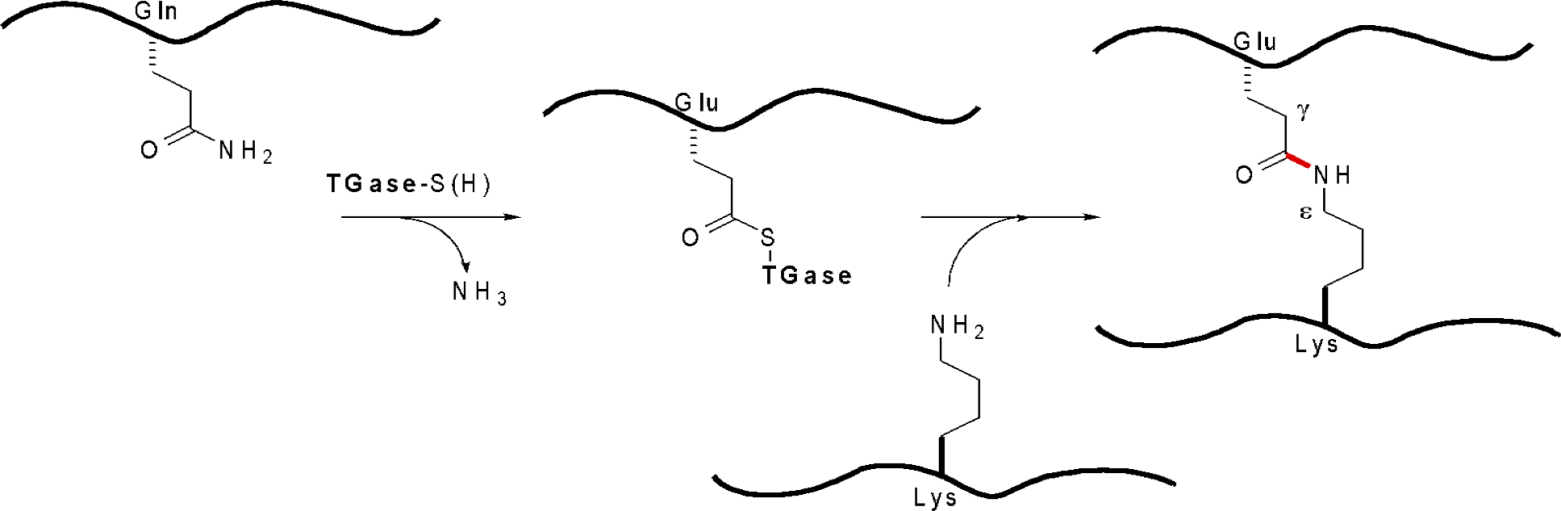
TGase-mediated protein cross-linking. Transamidation between protein-bound Gln and Lys residues leads to the formation of γ-glutamyl-ε-lysyl isopeptide bonds (red).

Transglutaminases (EC 2.3.2.13, amine-γ-glutamyltransferases) are a family enzymes that catalyze acyl transfer reactions in a Ca^2+^-dependent manner. Natively, TGase catalyzes the transamidation of the carboxamide moiety of a glutamine residue (acting as an acyl donor) with a primary amine, typically the side chain of lysine residue (acting as an acyl acceptor) resulting in protein cross-linking (Fig 1) [1]. The most commonly studied member of this family of enzymes is human tissue transglutaminase (TG2). As a ubiquitous enzyme whose biological role is context dependent, its unregulated activity has been associated with several disease states [2]. For example, TG2 has been identified as a contributor to cataract formation [1] and celiac disease [2], while increasing evidence suggests a link to autoimmune disease [3] and cancer metastasis [4]. TG2 is therefore considered a therapeutic target for many researchers.

TG2 has also been identified as a tool for *in vitro* site-specific protein labelling, given the short glutamine recognition sequence, its broad amine substrate specificity and its catalytic efficiency [5]. However, there are also drawbacks that limit the potential of this enzyme’s versatility as a tool for bio-conjugation, especially for intracellular applications. For example, opposing allosteric regulation by calcium and guanosine nucleotides (GDP/GTP) limits the conditions in which TG2-mediated labelling can occur [6]. Also, the endogenous expression of the ubiquitous enzyme must be considered when developing the biological application of any enzymatic labelling strategy. TG2 is also a relatively large protein, at 76 kDa [7], so concern may arise over functional interference during in-cell labelling.

The extended TGase family also includes bacterial TGases. Although they catalyze the same reaction as their mammalian counterparts, they bear very little structural resemblance to them. More importantly, bacterial transglutaminases are not restricted by the same calcium dependence and allosteric regulation as TG2 or other mammalian TGases [8]. Along with their broader substrate specificity and lower deamidation activity, bacterial transglutaminases have great potential as biocatalysts for protein labelling.

The most commonly studied bacterial TGase is the 38-kDa enzyme derived from *Streptomyces mobaraensis*, known as microbial transglutaminase (mTG). mTG has long been used in the food industry to act as a protein binding agent for meat and fish [9]. This is beneficial to the processing of different foods improving the texture and allowing the utilization of lower quality meats. Many reviews have been published on the application of mTG in the food industry [9].

The broad substrate specificity of mTG makes it amenable as a tool for conjugation and opens its potential as a biocatalyst. This functionality has been explored and studied in multiple reviews [10, 11]. However, mTG has one significant limitation that limits its functionality for *in cellulo* labelling. Natively, mTG is derived from *S*. *mobaraensis* as a zymogen bearing an N-terminal pro-peptide. The presence of the pro-peptide sequence has been hypothesized to facilitate the transport of the enzyme through the cytoplasm of a cell and into its medium [12]. More importantly, analysis of the structure of mTG suggests that the *N*-terminal pro-peptide sequence shields the active site of mTG, rendering the enzyme inactive while the peptide is bound. Given the promiscuity of mTG, it is hypothesized that the active form of the enzyme could be toxic to the host. It may be necessary to express the enzyme in an inactive form, in order to prevent the enzyme from cross-linking proteins to the detriment of cell viability [13]. To activate the original zymogen, *S*. *mobaraensis* also secretes two proteases that are responsible for the cleavage of the *N*-terminal pro-peptide [14]. Extensive research has gone into expressing mTG in yeast and *E*. *coli* [15, 16]; however, at the beginning of our investigation, expression of the enzyme in its active form [17] was not feasible. Although this constraint has not limited the use of mTG in the food industry [8, 9, 18], the recent shift of focus to bio-catalysis and bio-conjugation applications makes it clear that the requirement for mTG to be expressed in its active form represents a limitation.

A lesser known bacterial transglutaminase from *Bacillus subtilis* was discovered in 1996[19]. *B*. *subtilis* transglutaminase, also known as bTG, is implicated in the protection of the bacterium through the cross-linking of multiple coat proteins on the surface of a spore [19]. This multiprotein layer offers resistance against lytic enzymes and noxious chemicals, thereby enabling normal germination [20]. bTG expression levels have been shown to be directly proportional to the life cycle of the cell, with bTG expression increasing as cells begin to sporolate [20]. Therefore, bTG is characterized as part of a defense mechanism, designed to operate under adverse conditions [19, 20].

At 28 kDa, bTG is ~10 kDa smaller than mTG and shows little structural homology to its bacterial homologue. Moreover, the optimal temperature for bTG activity is 60 ºC, while it is 50 ºC for mTG, and the optimal pH for bTG is 8.2 while it is 6–7 for mTG [21, 22]. Despite these differences, we hypothesized that bTG probably maintains a broad substrate scope like other TGases, and that its robust activity may make it suitable for diverse applications as a biocatalyst [23, 24]. Additionally, bTG is natively expressed in its active form, suggesting that it may be the ideal TGase for intracellular labelling applications.

Other enzymes, including ligases and sortases [25, 26], have already been applied to intracellular labelling. Typically, a substrate peptide sequence is genetically fused to a protein of interest, prior to site-selective modification by the co-expressed enzyme. To apply bTG in this way, a substrate tag is required, bearing a glutamine residue in a sequence that shows high affinity for bTG. This would allow for site-selective transamidation, potentially with a wide variety of amines, as observed for TG2 [27] and mTG [28]. However, prior to this work, very little was known regarding the glutamine substrate specificity of bTG [29]. For other TGases such as mTG, TG2 and Factor XIII, high-affinity glutamine peptide sequences were identified by screening large phage-displayed libraries for activity as acyl-donor substrates [29–32]. Herein, we describe an alternative high-throughput approach that we used to identify the highest affinity glutamine sequences known for bTG. Furthermore, we demonstrate the functionality of these tags in proof-of-principle bioconjugation applications.

## Results and discussion

### In vitro FRET-based ligation assay

Intrinsically fluorescent proteins (FPs) are used extensively throughout molecular biology, as genetically encodable fluorophores that facilitate protein detection. As reviewed by Palmer, these diverse applications include the detection of protein-protein interactions in living cells [33]. Lee and co-workers recently studied the bTG-mediated ligation of FPs bearing substrate tags on their termini, using FRET to indicate as a proximity-based indicator [30]. More specifically, they used this methodology to screen a library of peptides as acyl-acceptor (lysine) substrates for bTG; herein, we adopted a similar approach to screen for acyl-donor (glutamine) substrate sequences.

**Fig 2:**
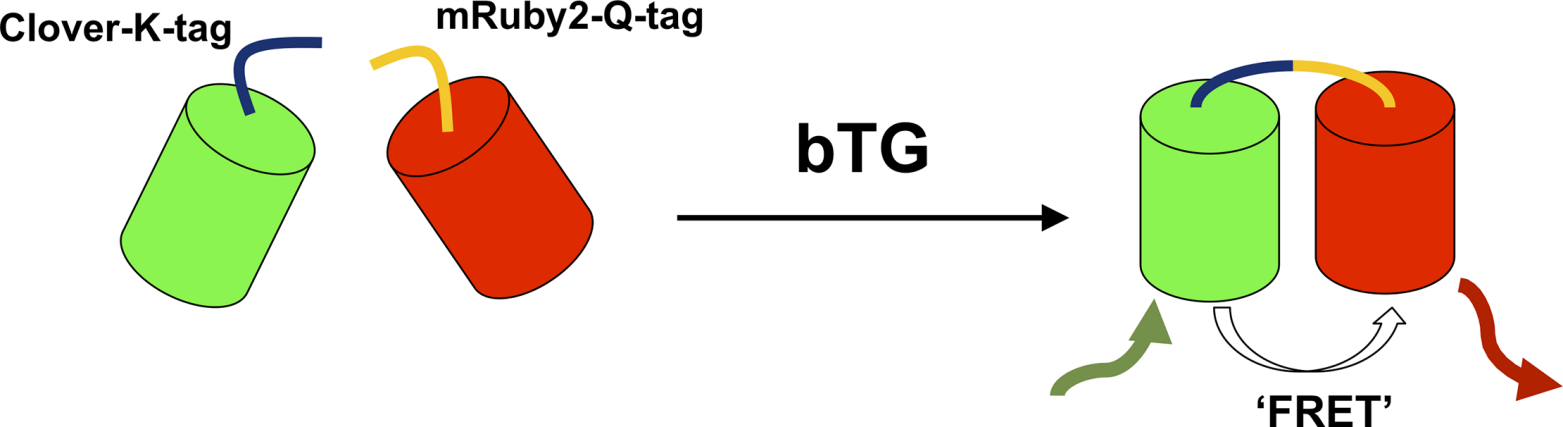
FRET-based peptide screening assay. Cartoon representation of the conjugation of mRuby2-Q-tag and Clover-K-tag, in the presence of bTG, resulting in a cross-linked product. Due to the spectral overlap of mRuby2 and Clover, when in close proximity, excitation of Clover leads to FRET, and red emission by mRuby2.

Fig 2 gives a pictorial representation of the FRET-based assay we developed to detect bTG-mediated cross-linking. In the presence of bTG, the glutamine sequence ‘Q-tag’ on the terminus of Clover is cross-linked to the lysine sequence ‘K-tag’ on the terminus of mRuby2, resulting in a fusion product containing FPs that were specifically engineered for spectral overlap [34]. This product can be detected by excitation of the FRET donor; due to the proximity of the two FPs, energy transfer leads to excitation of the FRET acceptor and its subsequent red emission. To apply this method to the screening of a library of different Q-tags, it was first optimized as a high-throughput assay.

**Fig 3:**
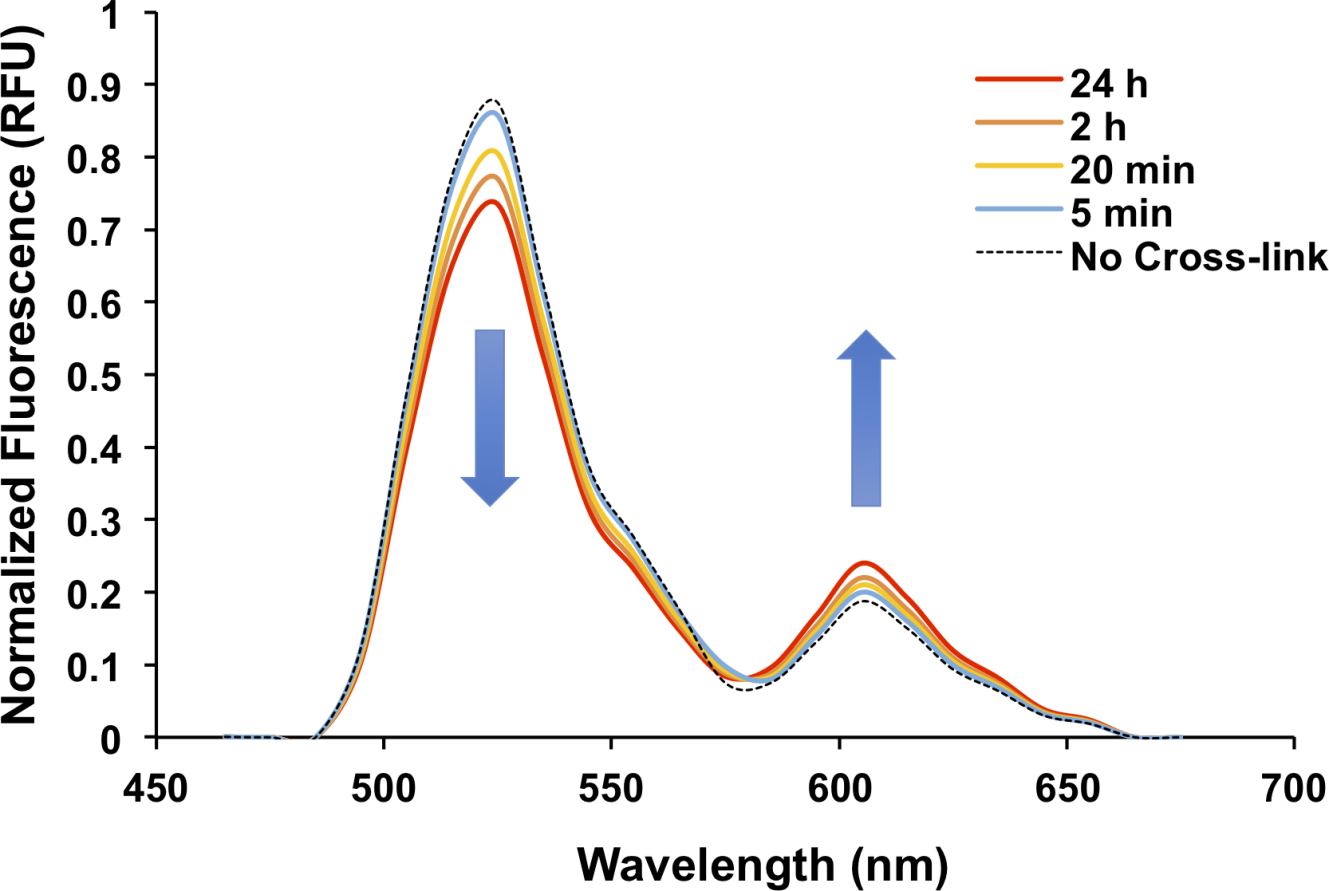
Emission spectrum scan of bTG-mediated *in vitro* conjugation of purified mRuby2-7M48 and Clover-6K. Samples were excited at 440 nm and scanned over a range of 450–700 nm at multiple time points (5 min to 24 h).

As a starting point for this optimization, we designed a Q-tag substrate and a K-tag substrate. As a generic K-tag, we chose the hexalysine sequence (aka 6K) previously shown to function as a bTG substrate[30]. For our initial Q-tag, we used a heptapeptide sequence (WLAQRPH, aka 7M48) previously shown to have high affinity for mTG [35]. The test proteins mRuby2-7M48 and Clover-6K were expressed and purified, and the bTG-mediated conjugation of these test proteins was then monitored by fluorescence spectroscopy through excitation at 440 nm and monitoring emission from 450–700 nm over a period of 24 hours. As shown in Fig 3, we observed a noticeable decrease in fluorescence emission in the green region of the visible spectrum, accompanied by an increase in the red emission, consistent with increased FRET efficiency in the sample. As a control for this experiment, an mRuby2-Clover fusion protein was designed that mimicked the spacer length between the expected mRuby2-Clover transamidation product (see Supporting Information, S1 File). The fusion control acts as the best possible result for the FRET assay, providing calibration for what a positive result should resemble. The FRET efficiency of the bTG reaction mixture over 24 h was still significantly lower than that of the mRuby2-Clover fusion protein; however, the time-dependent increase in FRET was encouraging, suggesting that the reaction could be used to test bTG reactivity with a given pair of Q- and K-tags.

### Design of peptide library for FRET-based screening

After confirming the FRET-based assay could be used to monitor bTG activity, a library of Q-tags was designed for screening. Comprehensive studies on the ideal length of peptide tag have not been conducted for bTG, but it is known that for mTG, a heptapeptide sequence represents a minimal tag that maintains affinity [36]. Given the limited information available, and the functionality of the 7M48 sequence, we chose to design a library of heptamer glutamine sequences (XXX-Q-XXX, where X is a variant residue) as Q-tags. In the design of this heptamer tag, the variation of all six X residues flanking the reactive glutamine with all 64 codons, covering all 20 amino acids, would result in library comprising over 68 × 10^9^ codons. To ensure 90% coverage of all possible sequence variants, over 158 × 10^9^ colonies would need to be screened [37, 38]. Although a flow cytometer can be used to screen this number of events, it is not obvious that a transformant library could capture this level of diversity, nor that it is necessary to create such a large library, for the purposes of identifying a reactive Q-tag. We reasoned that a smaller library comprising a subset of representative amino acids at each of the six flanking positions would still provide valuable information regarding substrate affinity, and would be amenable to analysis by a number of different diagnostic methods.

For this reason, we chose the ‘NMT’ degenerate codon as a means of coding for a limited range of amino acid residues. N represents all four base pairs, M represents thymine/guanine and T signifies thymine. This combination of nucleotides results in 8 codons that code for 8 amino acids: alanine (hydrophobic), asparagine (polar/uncharged), aspartate (negative/charged), histidine (positive/charged), proline (hydrophobic), serine (polar/uncharged), threonine (polar/uncharged) and tyrosine (aromatic). This restricted library represents all major amino acid types while keeping the library at a manageable size. Varying all six flanking residues with the NMT codon would result in a 262,144-codon library. To ensure 90% coverage of all possible peptide variants of this library, only ~6 × 10^5^ colonies would require screening.

### Controls for FRET-based screening by FACS

Once conditions for performing and monitoring *in vitro* FRET-based transamidation were established, *in cellulo* monitoring of transamidation was the next objective. This presented a different set of challenges to overcome, namely the expression and visualization of three separate proteins within one cell. A duet vector was used for the expression of both mRuby2-Q-tag and Clover-6K, to favour similar expression levels of the fluorescent protein substrates, as confirmed by SDS-PAGE (not shown). Plasmids for fluorescent protein substrates and bTG were transformed and expressed sequentially. pBAD24-bTG was chemically transformed into BL21-Gold(DE3) cells using ampicillin selection. After successful transformation, cells were harvested and made chemically competent. At this point, pACYCduet-1(mRuby2-Clover) was chemically transformed into the BL21-Gold(DE3)/pBAD24-bTG cell line using a kanamycin antibiotic marker. Details on the expression can be found in Materials and methods.

Upon adapting the FRET-based ligation assays to screening by FACS, we noted that excitation at 440 nm was not possible with the cytometer, so the cells to be sorted were excited using the 488 nm channel, the highest energy channel that allows monitoring of emission in both green and red channels. Controls were then performed to allow a distinction to be made between the direct excitation of mRuby2 (leading to ‘false positive’ red emission) and a FRET signal due to an authentic transamidation event. In total, five different controls were analyzed, as shown in the Supporting Information: 1) Clover alone, 2) mRuby2 alone, 3) the mRuby2-Clover fusion protein, 4) mRuby2-7M48 plus Clover-6K in the absence of bTG, and 5) mRuby2-7M48 plus Clover-6K in the presence of bTG (see S1 File). As described in detail in the Supporting Information (S1 File), the first control experiment confirmed that no fluorescence is detected in the red channel, in the absence of mRuby2. However, the second control confirmed that some red fluorescence is observed due to direct excitation at 488 nm, establishing an important ‘background’ signal for which we corrected. The third control served as a positive control for red fluorescence due to FRET (rather than direct excitation) from which the appropriate FRET-positive gating parameters were set for the FACS to follow. The fourth control showed that no FRET-positive events were detected in the absence of bTG, confirming that this signal is dependent on bTG activity. The fifth control confirmed that FRET-positive events were detected from cells co-expressing bTG and tagged fluorescent proteins that were confirmed to function as substrates (see Fig 3).

### Library screening using FRET-based FACS

Having established the sorting conditions, cells were transformed with the plasmid coding for bTG expression (under arabinose control) and with a duet vector coding for the IPTG-inducible expression of Clover-6K and mRuby2-6NMT-Q, where 6NMT-Q represents the degenerate XXX-Q-XXX Q-tag discussed above. The expression of bTG was induced and sorting analysis was performed periodically, 210–300 minutes later. We expected that at the shortest time points, we would observe FRET signal from the most reactive Q-tags, and that the population of cells displaying a FRET signal would increase over time, as the less efficient Q-tags were given time to react.

**Fig 4:**
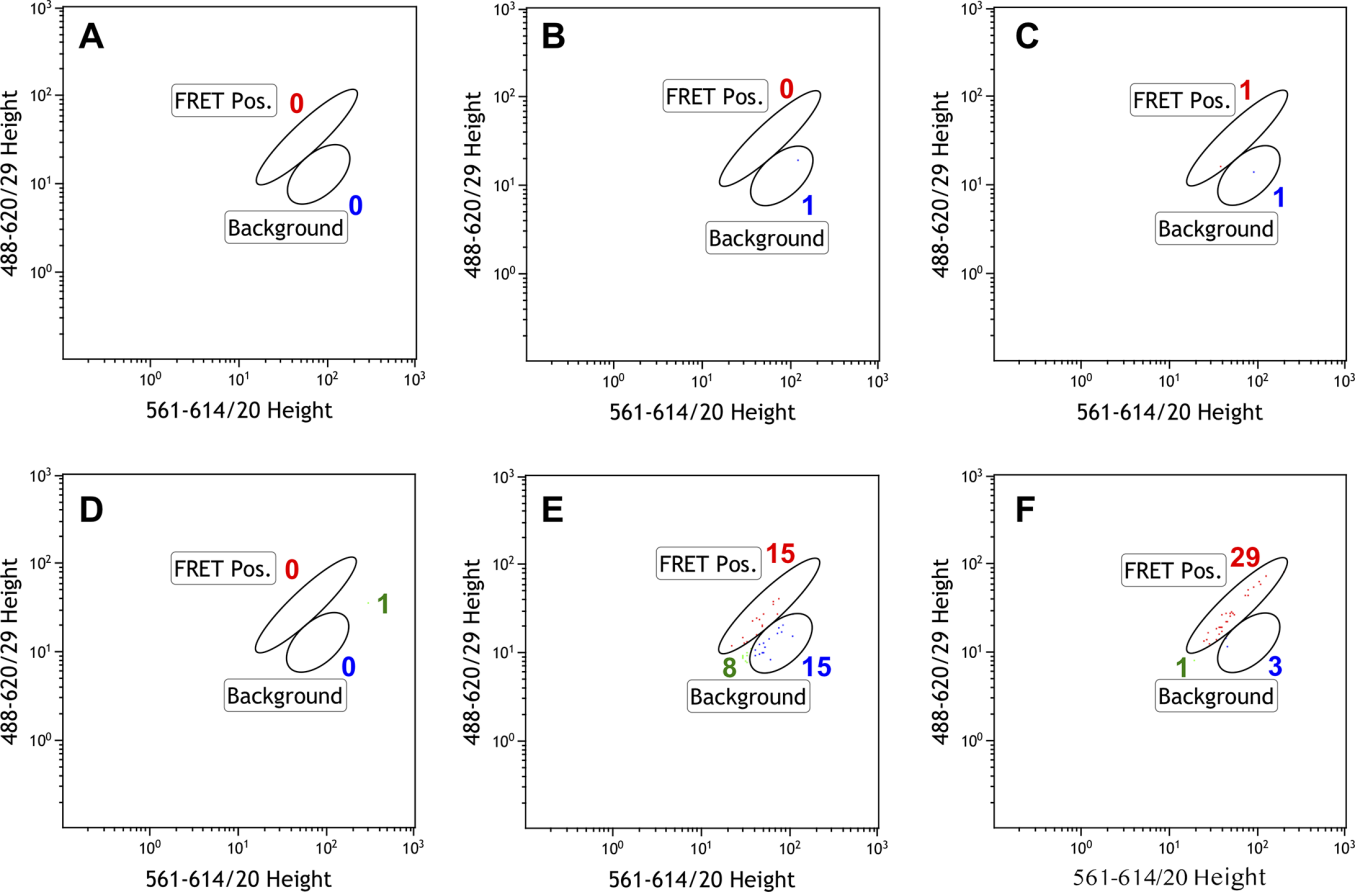
Flow cytometry analysis of cells expressing peptide library. FACS plots of BL21(DE3) Gold cells expressing mRuby2-6NMT-Q plus Clover-6K, with and without bTG, at time points corresponding to 210 min (A, D), 240 min (B, E) and 300 min (C, F) after bTG induction. Plots are gated for putative FRET cells (see S1 File), excitation at 561 nm, emission at 614 nm (red FP channel) vs. excitation at 488 nm, emission at 620 nm (FRET channel). Plots A-C are of cells that do not contain bTG; plots D-F are of cells in which bTG expression has been induced. Gated cells and numbers of sorting events are colour-coded to identify distributions of populations from plot to plot (green = putative FRET, red = FRET positive, blue = background red fluorescence).

When cells were sorted 210 min after induction of bTG expression, no events were observed in the gated FRET-positive area, as shown in Fig 4D. As a control, cells that did not express bTG were also sorted, to ensure that any background FRET signal would not give rise to a false positive result. As Fig 4A shows, no events were found in the FRET-positive region among cells not expressing bTG, either. The results obtained 240 min after bTG induction suggested that as time elapsed, there was an increase in the number of FRET-positive cells, as shown in Fig 4E. This indicated that the assay is capable of detecting an increase in the concentration of transamidation product within the cell, so these cells were collected for further analysis. When cells were screened 300 min after bTG induction, a significantly increased number of events were detected in the gated FRET-positive region of the plot (Fig 4F). Furthermore, the same gated region was void of such events in the cells that did not express bTG (Fig 4C), confirming that we were detecting bTG-mediated ligation product. These cells were also retained for further characterization.

### Identification of reactive Q-tag sequences

**Fig 5:**
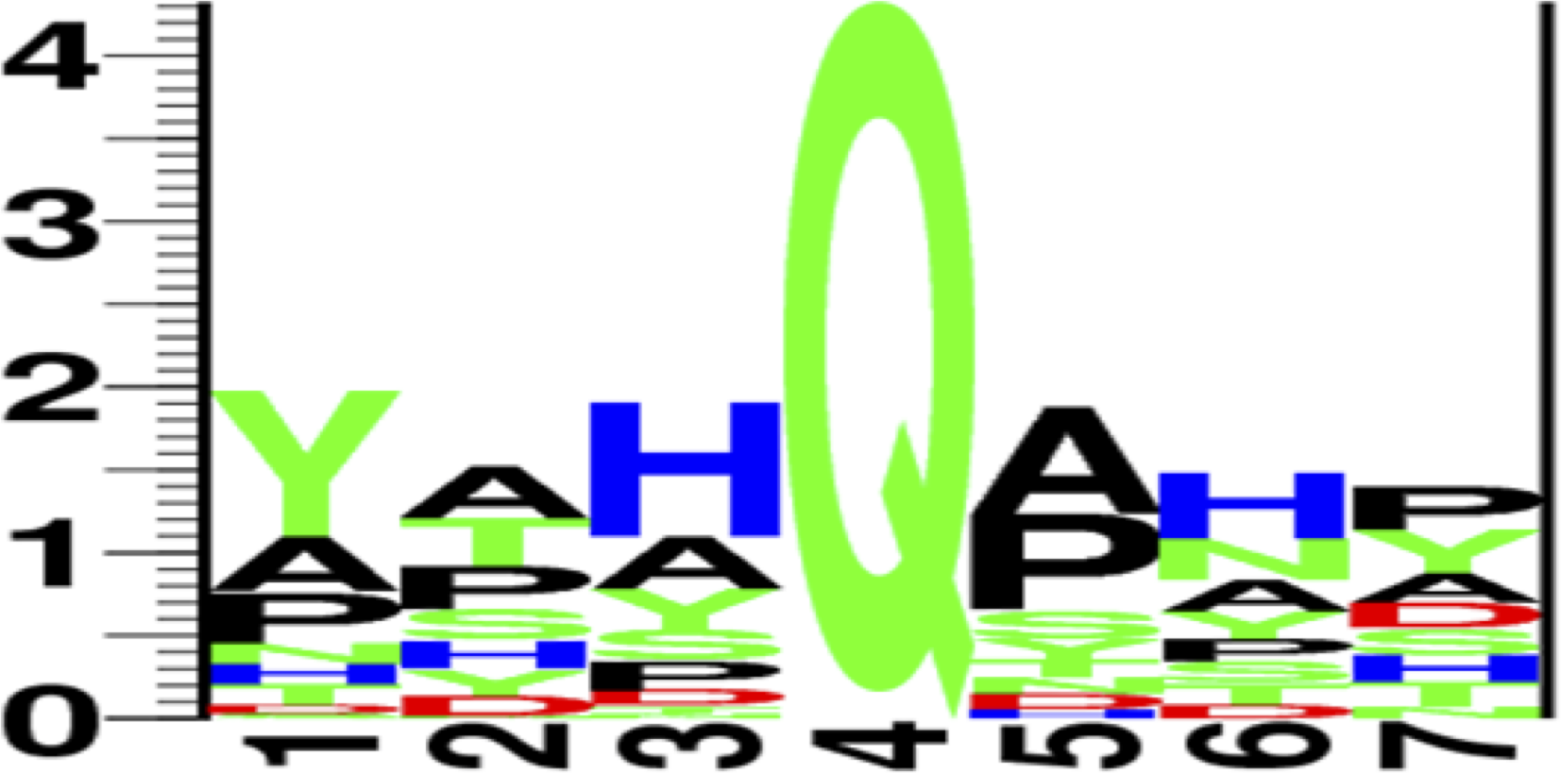
Protein logo of peptide library sequencing results. Protein logo representing the sequencing results for 78 sequences from the FRET-positive fraction of the 300-min mRuby2-6NMT-Q library. (http://rth.dk/resources/plogo/).

While some FRET-positive cells were detected 240 min post-induction, only those collected after 300 min were numerous enough to provide a density of viable cells that could be re-cultured for characterization. These cells were plated and of the colonies grown, 94 were picked and re-grown in a 96-well plate. This plate was then subjected to DNA extraction and sequencing, resulting in 78 successfully sequenced Q-tags (see Supporting Information, S2 File). Structurally, the most common types of residues to appear at each position are: 1- aromatic, 2- aliphatic/aromatic, 3- basic, 4- glutamine, 5- aliphatic, 6- basic and 7- aromatic/aliphatic. Fig 5 depicts a protein logo of the sequencing results, representing the frequency at which a given amino acid appears at each position.

In a more detailed analysis of the most obvious sequence patterns, of the 78 sequenced Q-tags, 35 of them (45%) had a tyrosine at position 1. This high frequency suggests that bTG may have an affinity for aromatic amino acids within the glutamine substrate binding site. At position 2, 16 sequences (21%) presented an alanine residue. At position 3, a histidine residue was present in 33 sequences (42%). Sixteen sequences (21%) had Y1/H3, and among these, 7 (44%) had an alanine residue at position 2, which is significantly higher than the frequency of A2 among all the sequences. This suggests that in the Y1/H3 sequence there is a significant preference for alanine at position 2. The sequence Y1-A2-H3 occurred 7 times out of 78 sequences (9%), which is significantly higher than one would expect, based solely on the combined probability of each residue at each position, which would have given 4%.

Considering the residues flanking the central glutamine, of the 33 sequences presenting H3, 15 of them (45%) also presented and alanine at position 5, which is relatively more frequent than the 27 occurrences of alanine at this position among all 78 sequences (35%). The 15 occurrences of H3-Q4-A5 among the 78 sequences (19%) is slightly higher than what one would expect based on the relative frequencies of H3 and A5 alone (15%). Alternatively, P5 occurs in 24 of the 78 sequences (31%) and in 11 of the 33 H3 sequences (33%), suggesting there may not be a significant preference for the H3/P5 combination. Although there is a structural similarity between the two sub-sequences (proline and alanine both being hydrophobic), the conformational flexibility afforded to a peptide with alanine over proline can result in very different binding mode and affinity.

Considering positions 5–7, 27 sequences presented an alanine residue at position 5 (35%), while 24 sequences presented a proline at this position (31%). At position 6, a histidine residue was the most common, occurring 21 times (27%). At position 7, proline occurred 15 times (19%) and tyrosine occurred 14 times (18%). Interestingly, of the 21 H6 sequences, as many as 11 also had Y7 (52%), well above the relative frequency of tyrosine at that position overall. The sequence H6-Y7 therefore occurred 11 times out of 78 sequences (14%), which is well above the relative frequency expected based solely on the combined frequency of each residue (5%). This suggests the H6-Y7 motif may show enhanced affinity through cooperativity. On the other hand, the 5 occurrences of A5-H6-Y7 (45% of the 11 H6-Y7 sequences) and the 4 occurrences of P5-H6-Y7 (36%) are only slightly higher than the relative frequency of A5 and P5 among all 78 sequences. This suggests the binding of the H6-Y7 motif may not confer a preference for a particular residue at position 5, at least among our limited library.

From the sequence analysis of the most reactive Q-tags selected at the 300-min time point, several were chosen for subsequent kinetic characterization (Table 1). The peptide sequence YAHQAHY is the only one that was observed three times among the 74 sequences, while no other sequence was found more than once. Three other similar sequences, analogous to the structural trend noted above, were also chosen for comparison. As a control, two other peptides (YAHQAAH and YSHQAHY) were chosen that did *not* display all the trends possessed by the first four sequences. These proteins were then tested as bTG substrates using the GDH-coupled assay previously adapted for use with TG2 [39] and mTG [35]. Specificity constants were measured with this assay (Table 1) in order to compare the relative reactivity of the Q-tags.

**Table 1:** Specificity constants of TGase-mediated transamidation. 0.2–0.8 μM mRuby2-Qtag test proteins and 5 mM Gly-OMe were reacted in the presence of 0.1 U TGase. Initial rates were measured over 20 min, using the GDH-coupled assay. Specificity constants were determined by fitting initial rate data to the Michaelis-Menten equation (see Materials and methods).

Protein substrate	k_cat_/K_M_ (μM^-1^ min^-1^)		
	bTG	mTG	TG2
mRuby2-YAHQAHY	19 ± 3	34 ± 3	7 ± 1
mRuby2-YAHQPHY	13 ± 1	4.0 ± 0.2	4.0 ± 0.2
mRuby2-YPHQPHY	5 ± 2	3 ± 1	8 ± 5
mRuby2-YPHQAHY	8 ± 2	13 ± 3	1.0 ± 0.5
mRuby2-YSHQAHY	4 ± 1	0.20 ± 0.04	7 ± 2
mRuby2-YAHQAAH	2 ± 1	29 ± 2	2 ± 1
mRuby2-7M48	7 ± 1	41 ± 4	9 ± 2
MBP-RTQPA	3.0 ± 0.4	7 ± 3	0.30 ± 0.04
MBP-RLQQP	1.0 ± 0.1	6 ± 2	1.0 ± 0.5

As can be seen from Table 1, the peptide sequence that appeared the most frequently among the selected Q-tags (mRuby2-YAHQAHY) also showed the highest *in vitro* reactivity with bTG, followed by the analogous YAHQPHY tagged protein. By way of comparison, two previously reported [30] reactive glutamine sequences were constructed and genetically fused to the Maltose Binding Protein (MBP) as a test protein. Of these two test proteins, MBP-RTQPA was 6-fold less reactive than mRuby2-YAHQAHY, and MBP-RLQQP, which contains two potentially reactive glutamines, was 16-fold less reactive. The greater kinetic efficiency of the best tags discovered through our peptide library screen validates our screening method and firmly establishes YAHQAHY and YAHQPHY as an advanced starting point for the study of bTG substrate specificity.

As an additional consideration, specificity constants were tested for mTG and TG2 as well. For the end goal of applying bTG to in cellulo protein labelling, it is important that the selected tag is selective for bTG and shows limited reactivity with TG2, a ubiquitous enzyme within mammalian organisms [40]. As shown in **Table 1,** most of the peptide sequences show widely varying levels of reactivity among the TGases tested. TG2 showed the greatest efficiency in its reaction with 7M48, YPHQPHY and YSHQAHY. However, both 7M48 and YAHQAHY react significantly more efficiently with their intended TGase (mTG and bTG, respectively) than they do with TG2. It should be noted that in the course of this initial study, we screened for reactivity with bTG, and not necessarily for orthogonality to TG2.

Since we performed these experiments, another TGase has been reported [41], allowing us another point of comparison. The microbial TGase from *Kutzneria albida* (KalbTG) was expressed as a soluble 26-kDa enzyme, and a highly specific glutamine substrate sequence, namely GGGYRYRQGGGG, was engineered for the bioconjugation application of KalbTG. The authors reported a K_M_ value of 2000 μM for their engineered peptide, and a k_cat_ value of 115 min^-1^, leading to a k_cat_/K_M_ value of 0.053 μM^-1^min^-1^, nearly 350-fold lower than the value we measured for bTG reacting with the YAHQAHY tag (Table 1). This may be due in part to the millimolar K_M_ value reported for the GGGYRYRQGGG tag, which illustrates that its substrate binding efficiency does not have to be high, for it to be very selective for KalbTG over mTG.

### Proof of principle bioconjugation

To investigate the potential of using bTG to label a protein of interest (POI), four mRuby2 proteins bearing highly reactive Q-tags–namely, YAHQAHY, YAHQPHY, YPHQAHY and YPHQPHY were selected for further study (hereby referred to as BQ1, BQ2, BQ3 and BQ4, respectively). Dansyl-cadaverine was chosen as an amine substrate for direct incorporation into the tagged-mRuby2 variant proteins, due to the structural similarity between the cadaverine amine and the side chain of lysine. The initial rates of transamidation between Q-tagged mRuby2 and dansyl-cadaverine were measured using the GDH-coupled assay [35]. As shown in Table 2, the BQ1-tagged test protein showed the highest reactivity, as expected. Importantly, when mRuby2 (with no fused Q-tag) was studied as a negative control, no reaction was observed, confirming the specificity of the transamidation reaction.

**Table 2:** Initial rates of bTG-mediated transamidation of POI-Q and dansyl cadaverine. The reaction of 0.8 μM of Q-tagged mRuby2 test protein with 5 mM dansyl cadaverine was mediated with 0.1 U of bTG over 20 min and detected using the GDH-coupled assay (see Materials and methods).

POI-Qtag	Initial Rates (μM/min)
mRuby2-BQ1	12.2 ± 0.5
mRuby2-BQ2	9.1 ± 0.4
mRuby2-BQ3	1.9 ± 0.5
mRuby2-BQ4	6.7 ± 0.2
mRuby2	not detected

**Fig 6:**
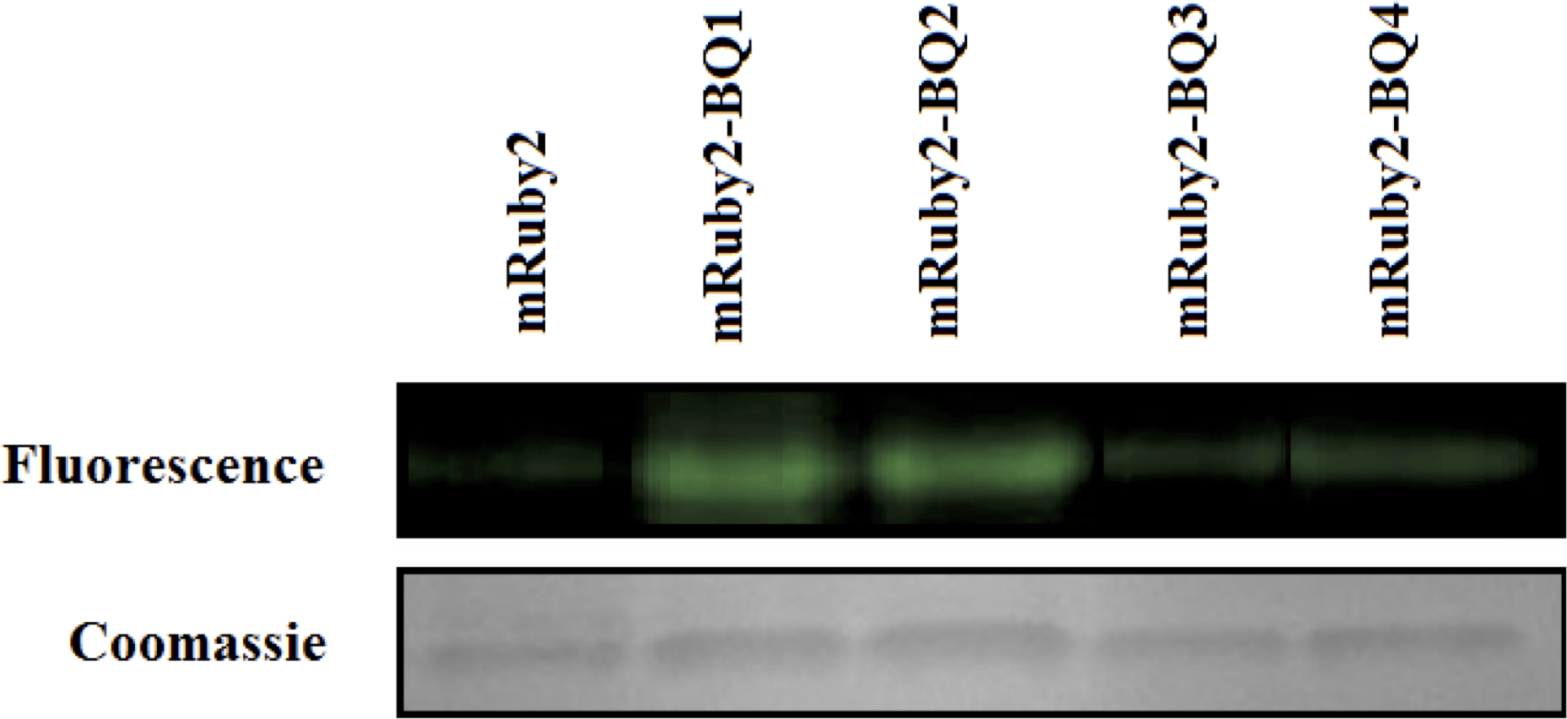
bTG-mediated fluorescent labelling of mRuby2 bearing high-affinity Q-tags. Purified test proteins bearing bTG recognition tags were fluorescently labelled with dansyl-cadaverine through bTG-mediated transamidation. SDS-PAGE gels of test proteins were run, followed by irradiation to visualize any fluorescent bands. After fluorescent visualization, Coomassie staining was performed to confirm the presence of protein bands.

The products of these bTG-mediated labelling reactions were also studied qualitatively. After labelling 0.02 mg/mL of Q-tagged test proteins with 5 mM dansyl-cadaverine in the presence of 0.1 U bTG over 3 h at 37 ºC, the product mixtures were analysed by SDS-PAGE. As shown in Fig 6, the fluorescent labelling of the most reactive Q-tagged test proteins was easily visualized. To the best of our knowledge, this is the first time bTG has been used to label a protein. As expected, a significantly more fluorescent band was observed for mRuby2 test proteins bearing the highly reactive BQ1 or BQ2 tags on their terminus. As a control, fluorescent labelling was attempted using untagged mRuby2. Importantly, no fluorescent band was detected, suggesting that no bio-conjugation occurred in the absence of a Q-tag. This is an important observation, suggesting that no reaction is possible in the absence of a reactive glutamine residue, and that the labelling of a reactive Q-tag is site-selective.

## Conclusions

In summary, a peptide library was designed and screened with a FRET-based ligation assay in order to identify highly reactive glutamine substrate sequences of bTG. The new sequences identified were confirmed kinetically to be the most efficient Q-tags ever reported for bTG. Subsequently, bTG-mediated fluorescent labelling of a protein was demonstrated for the first time.

Given the stringent parameters applied to the size of the library screened herein, it is conceivable that in the future, a more exhaustive library containing all 20 amino acids at the variant positions could be screened. Furthermore, it would be valuable to measure the amine (acyl-acceptor) substrate scope of bTG, in order to know how bTG could be used most effectively in protein labelling contexts, potentially including its application in living cells.

## Materials and methods

### FRET-based assay optimization

Expression and purification of tagged fluorescent proteins and mTG/bTG was performed according to the detailed description found in the Supporting Information (S1 File). 0.8 μM of mRuby2-7M48 and Clover-6K were incubated in 1 mL reaction mixture, buffered at pH 7.2 with 40 mM MOPS. The sample was excited at 440 nm and scanned from 440–700 nm to identify the baseline of emission before cross-linking had occurred. This was followed by the addition of 0.1 U of mTG into the reaction mixture. Spectral scans were run on the reaction over the next 24 h using a plate reader. 200-μL samples were scanned at 37 ºC with no shaking.

### In cellulo expression of mRuby2, Clover and bTG

The cloning and expression of bTG and the assay proteins is described in the Supporting Information (S1 File). Prior to sorting, 100-mL cultures were inoculated with a 5-mL pre-culture. Cultures were grown to an OD of 0.3 at 37 ºC. At this point, an IPTG induction (final concentration 400 μM) was carried out for 3 hours at 28 ºC. After the induction period, cells were lightly pelleted (2000 rpm, 30 min) and re-suspended in 100 mL of fresh LB. Cells were then incubated at 4 ºC, for 24–36 hours (to allow for the slow maturation of fluorescent proteins, especially mRuby2). Afterwards, cultures were incubated at 28 ºC prior to induction of bTG expression using 5% arabinose. Expression in sub-optimal conditions required the higher concentration of arabinose for induction. Five-millilitre aliquots were then taken at different time points (including 210 min, 240 min, and 300 min after induction) and cooled to 4 ºC to halt transamidation activity prior to flow cytometry. The co-expression of the three proteins and the effective cross-linking of the substrates, was confirmed by SDS-PAGE of a reaction mixture containing mRuby2-7M48, Clover-6K and bTG, expressed within *E*. *coli* after 24 h. As a control, a culture that did not undergo arabinose induction was also analysed, showing no enzyme nor cross-linked proteins.

### FACS screening

Bacterial cells (including controls and those expressing bTG and/or tagged FP substrate proteins) were pelleted by centrifugation, prior to aspiration of LB medium, washing with sheath buffer (IsoFlow™ Sheath Fluid, Beckman Coulter, Miami, FL) to remove residual LB medium, and re-suspension in 1 mL sheath buffer for sorting. Cells were sorted using a high-speed cell sorter (MoFlo Astrios EQ, Beckman Coulter, Miami, FL). Forward and side scatter (FSC and SSC) voltage parameters for the bacterial cell samples were adjusted with the aid of polystyrene size calibration beads (MegaMix-Plus FSC, Biocytex, Marseille France) with 100-nm, 300-nm, 500-nm, and 900-nm size populations. For the FACS sorting experiment, cells were selected on the basis of high fluorescence intensity resulting from successful FRET based ligation detected through excitation at 488 nm (mClover excitation) and fluorescence emission detection through 614/20 bandpass and 620/29 bandpass filters (mRuby2 emission). To enhance the stringency of the cell screening process the “Single Mode” setting was used to ensure only drops exhibiting a single event were selected.

### Sequencing preparation

After cells were collected from cell sorter, colonies were grown on LB/chloramphenicol plates overnight (16 h) at 37 ºC. Ninety-four colonies were then picked and re-grown in 200 μL of LB with 25 μg/mL of chloramphenicol within a standard 96-well plate, overnight at 37 ºC. After incubation, DNA extraction and sequencing were performed by Eurofins Scientific.

### Expression and purification of Q-tagged MBP-mRuby2 variants from library

Pre-cultures of cell-stocks from selected FRET-positive cells were prepared using 5 mL of LB containing 100 μg/mL ampicillin and 0.2% glucose. MBP-mRuby2 constructs were expressed using established MBP expression protocols. To separate mRuby2 from the Clover, affinity chromatography was used to purify MBP-mRuby2-Q. Only the MBP-mRuby2-Q proteins possess a Hex-His tag that can be used for additional purification. The lysate was purified using 1 mL of Ni-NTA resin, equilibrated in 50 mM phosphate buffer (pH 8.0) with 300 mM NaCl and eluted with an imidazole gradient (50 mM phosphate buffer, pH 8.0, with 300 mM NaCl 140 mM imidazole) on a gravity column. The purified activated MBP-mRuby2-Q was dialyzed against 50 mM phosphate buffer (pH 8.0). Protein concentration was quantified using the Bradford protein assay.

## Supporting information

S1 File Fluorescence spectra, gating controls for FACS screening, protein expression and purification procedures, and cloning procedures. (DOCX)

S2 File Substrate sequences in table format, facilitating analysis of trends. (XLSX)
