# Supplementary material for: Design of a glutamine substrate tag enabling protein labelling mediated by Bacillus subtilis transglutaminase
Source: PLoS One. 2018 May 30;13(5):e0197956. doi: 10.1371/journal.pone.0197956 (PMC5976192; doi:10.1371/journal.pone.0197956)
Supplement: S1 File — (DOCX) [file pone.0197956.s001.docx]

**Supporting Information for**

**Design of a Glutamine Substrate Tag**

**Enabling Protein Labelling Mediated by**

***Bacillus subtilis* Transglutaminase**

Samuel K. Oteng-Pabi, Christopher M. Clouthier and Jeffrey W. Keillor*

*Department of Chemistry and Biomolecular Sciences*

*Centre for Catalysis and Research Innovation*

*University of Ottawa*

*30 Marie-Curie, Ottawa, Ontario*

*K1N 6N5 Canada*

Table of Contents

mRuby2-Clover fusion protein FRET spectrum 2

Gating controls for screening by FACS 4

bTG cloning, expression and purification 11

Cloning and expression of mRuby2 and Clover into pACYCduet-1 13

# mRuby2-Clover fusion protein FRET spectrum

As a control for this experiment, an mRuby2-Clover fusion protein was designed that mimicked the spacer length between the expected mRuby2-Clover transamidation product. Given that all the Q-tagged proteins designed in this study include a GSSGSS spacer, and that reactive glutamine residues were to be placed at position four in the heptapeptide tag, the fusion product was designed with a 19-residue spacer between the two FPs. This fusion control thereby mimics the cross-linked product from the bTG-mediated cross-linking reaction. Its FRET response also sets the bar for what a positive result would resemble, ideally, from the FRET-based screening assay. It is evident from Fig S1 that the positive control gives a strong FRET signal (excitation in the green, emission in the red) but also shows significant green emission. This would suggest that the proximity and orientation of the FPs may not perfect, but still allow significant FRET to occur.

Although the FPs selected have minimal overlap between their excitation bands, there is still a concern that the limited direct excitation of mRuby2 may result in false positives that can distract from the true results of the experiment. To address this issue, we explored the excitation of Clover at a lower wavelength, below its maximum. While this would reduce the intensity of the overall signal observed, it would also eliminate any direct excitation of mRuby2, reducing false positive results. This resulting spectrum is also shown in Fig A. Excitation of Clover at 440 nm reduces the overall signal of red emission but ensures the signal is based on FRET and not direct excitation of mRuby2.

**Fig A: Emission spectra of mRuby2-Clover fusion protein.** Excitation of the fusion protein at the Clover excitation maximum, 500 nm, resulted in greater red and green emission. Excitation at the blue end of Clover’s excitation band reduced the overall emission but still resulted in significant red emission due to FRET.

# Gating controls for screening by FACS

**1) Clover alone**

In the first control, involving cells expressing only Clover, no transamidation was possible. FACS analysis of these cells, resulting only in green emission upon excitation at 488 nm, is shown in Fig B. This control assures us of the lack of red emission in cells that do not express red FP.

**Fig B. Flow cytometry analysis of cells expressing Clover fluorescent protein.** FACS plots of BL21(DE3) Gold cells expressing the Clover fluorescent protein. 1) Ungated forward and side scatter, to distinguish bacterial populations from other submicron particulate, based on the size and granularity of the event. 2) Gated for bacteria, excitation at 488 nm, emission at 620 nm (FRET) vs excitation at 488, emission at 513 nm (green FP). 3) Gated for bacteria, excitation at 561 nm, emission at 614 nm (red FP) vs excitation at 488 nm, emission at 513 nm (green FP). Gated cells are colour-coded to identify distributions of populations from plot to plot (grey = total bacteria population, green = Clover-positive cells, blue = Clover-negative cells). Note the lack of red fluorescence in these cells, which do not express red FP.

**2) mRuby2 alone**

In the second control, the same channels used to gate and sort cells expressing Clover (see above) were applied to cells expressing mRuby2 alone (see Fig C). Importantly, this control showed a population of cells (x-axis, Fig C2) detected in the ‘FRET channel’ (excitation at 488 nm, emission at 620 nm) that are in fact events due to direct excitation of mRuby2 and not due to FRET. This ‘false positive’ provides a background reading for the FRET channel that was carefully accounted for throughout the screening.

**Fig C. Flow cytometry analysis of cells expressing mRuby2 fluorescent protein.** FACS plots of BL21(DE3) Gold cells expressing the mRuby2 FP. 1) Ungated forward and side scatter, to distinguish bacterial populations from submicron particulate background. B) Gated for bacteria, excitation at 488 nm, emission at 620 nm (FRET channel) vs. excitation at 488, emission at 513 nm (green FP channel). C) Gated for bacteria, excitation at 561 nm, emission at 614 nm (red FP channel) vs. excitation at 488 nm, emission at 513 nm (FRET channel). Gated cells are colour-coded to identify distributions of populations from plot to plot (grey = total bacteria population, red = mRuby2-positive cells, blue = mRuby2-negative cells). Note the significant population of cells along the x-axis of panel 2, due to the direct excitation of mRuby2, which provides a ‘background’ value for the FRET channel.

**3) mRuby2-Clover fusion protein**

In Fig D the same gating methods established with the first two controls were applied to cells expressing the mRuby2-Clover fusion protein. Importantly, a new population of events was detected in the upper right quadrant of Fig D2, consistent with cells that express the fusion protein *and* exhibit red emission in the FRET channel. Further analysis in Fig D3 allowed us to separate these events into two distinct populations. Both populations exhibit similar intensity when analysed in the red FP channel (x-axis), consistent with these cells expressing mRuby2. However, one population shows distinctly greater intensity along the y-axis (FRET channel), consistent with cells that express a fusion protein that exhibits an enhanced FRET signal. Furthermore, the population showing less intensity along the y-axis (FRET channel) is also found along the x-axis of panel B, which was previously identified as cells undergoing emission due to direct excitation of mRuby2 (see above). Presumably, the observation of a population of cells that exhibit red fluorescence and no apparent FRET signal may indicate that the translation and/or folding of the large fusion protein may be problematic, resulting in some cells that only express the *N*-terminal mRuby2 portion as a properly folded, functional FP.

**Fig D. Flow cytometry analysis of cells expressing mRuby2-Clover fusion fluorescent protein.** FACS plots of BL21(DE3) Gold cells expressing the mRuby2-Clover fusion fluorescent protein. 1) Ungated forward and side scatter, to distinguish bacterial populations from other submicron particulate. 2) Gated for bacteria, excitation at 488 nm, emission at 620 nm (FRET channel) vs. excitation at 488, emission at 513 nm (green FP channel). 3) Gated for Putative FRET cells, excitation at 561 nm, emission at 614 nm (red FP channel) vs. excitation at 488 nm, emission at 620 nm (FRET channel). Gated cells are colour-coded to identify distributions of populations from plot to plot (grey = bacteria, green = putative FRET, red = FRET-positive cells, blue = background). Note the distinct separation between populations of authentic FRET-positive cells (red) and those that show some red fluorescence due to direct excitation of mRuby2 (blue).

**4) Co-expression of substrate proteins mRuby2-7M48 and Clover-6K**

The fourth control featured the co-expression of mRuby2-7M48 and Clover-6K in the absence of bTG. This represents a negative control; without bTG, transamidation will not occur and red emission due to FRET should not be detected. Cells expressing both of these FP substrates were sorted and analysed as for the fusion protein, the results of which are shown in Fig E. Very few cells were found in the upper right quadrant of panels 2 and 3, suggesting limited FRET was occurring in the absence of covalent cross-linking.

**Fig E. Flow cytometry analysis of cells expressing mRuby2-7M48 + Clover-6K fluorescent proteins.** FACS plots of BL21(DE3) Gold cells expressing mRuby2 + Clover fluorescent proteins, cultured for three hours after maturation of fluorescent proteins. 1) Ungated forward and side scatter, to establish bacterial populations vs. other submicron particulate. 2) Gated for bacteria, excitation at 488 nm, emission at 620 nm vs. excitation at 488, emission at 513 nm. 3) Gated for putative FRET cells, excitation at 561 nm, emission at 614 nm vs. excitation at 488 nm, emission at 620 nm. Gated cells are colour-coded to identify distributions of populations from plot to plot (grey = bacteria, green = putative FRET, red = FRET-positive cells, blue = background). Note the limited number of cells that exhibit red emission due to putative FRET (panel B); presumably those identified in the upper right quadrant of panel 3 are due to ‘background’ FRET due to non-specific approximation.

**5) Co-expression of substrate proteins and bTG**

Finally, cells co-expressing mRuby2-7M48 and Clover-6K in the presence of bTG were sorted. Since we had previously demonstrated that the substrate proteins mRuby2-7M48 and Clover-6K could be cross-linked with by bTG *in vitro*, leading to a detectable FRET signal, these cells served as a positive control for FACS. Similar to what was observed with the fusion protein control, a significant population of putative FRET cells was detected (upper right quadrant of Fig F2), 3 h after adding arabinose to induce expression of bTG. Furthermore, this population was shown to be distinct from those exhibiting red fluorescence through direct excitation of mRuby2 (Fig F3).

**Fig F. Flow cytometry analysis of cells expressing mRuby2-7M48 + Clover-6K + bTG**. FACS plots of BL21(DE3) Gold cells expressing mRuby2-7M48 plus Clover-6K substrates, in the presence of bTG. 1) Ungated forward and side scatter, to distinguish bacterial populations from other submicron particulate. 2) Gated for bacteria, excitation at 488 nm, emission at 620 nm (FRET channel) vs. excitation at 488, emission at 513 nm (green FP channel). 3) Gated for putative FRET cells, excitation at 561 nm, emission at 614 nm (red FP channel) vs. excitation at 488 nm, emission at 620 nm (FRET channel). Gated cells are colour-coded to identify distributions of populations from plot to plot (grey = bacteria, green = putative FRET, red = FRET-positive cells, blue = background). bTG expression was induced for 3 h prior to analysis. Note the significant population of FRET-positive cells, distinct from those exhibiting red fluorescence due to direct excitation of mRuby2, demonstrating the ability of bTG to cross-link the substrates mRuby2-7M48 and Clover-6K, within 3 h of induction.

Taken together, these controls allowed us to determine the gating parameters necessary to isolate cells showing an enhanced FRET signal, due to bTG-mediated transamidation, as a population distinct from those exhibiting red fluorescence from ‘background’ direct excitation.

# bTG cloning, expression and purification

Forward and reverse primers were synthesized by IDT. Primers were designed that cloned the *tgI* gene from the genomic DNA of *B. subtilis*. Using Touchdown PCR, the *tgI* gene was amplified into the pBAD24 vector, which permits arabinose induction of gene expression .

**Table A. Oligonucleotides used for cloning bTG from *Bacillus subtilis***

| pBAD_bTG_Fw_NdeI | 5’ - CGC CAT ATG ATG ATT ATT GTA TCA GGA CAA T - 3’ |
| --- | --- |
| pBAD_bTG_Rv_NcoI | 5’ - CCC CCA TGG TTA ATG GCG GAC GAT GCG G - 3’ |
| pBAD_bTG_6His_Rv_NcoI | 5’ - CCC CCA TGG TTA ATG ATG ATG ATG ATG ATG ATG GCG GAC GAT GCG G - 3’ |

The PCR was performed in a BioRad® thermal cycler. After amplification, the gene was digested and ligated into the pBAD24 vector. The constructed vector was then transformed into chemically competent *Escherichia coli* BL21-Gold (DE3) in the presence of 100 µg/mL ampicillin by heat shock. Gene inclusion and reading frame were verified through sequencing. For sequence confirmation, pBAD24-bTG-6His was extracted and analyzed using restriction digest as well as sequence verification from *Eurofins Scientific.*

The pBAD24-bTG plasmid encoding for bTG bearing a *C*-terminal hexa-histidine tag was transformed in to *E. coli* BL21(DE3) in the presence of 100 µg/mL ampicillin. A 5-mL pre-culture sample was grown overnight and used to inoculate a 1-L culture that was grown to an OD of 0.3 before induction with 0.5% L-arabinose for 2 h. The culture was centrifuged at 3000 × g for 30 min at 4 ºC; the supernatant was discarded and the pellet was resuspended in 40 mM phosphate buffer pH 8.0, with 250 mM NaCl. Cells were disrupted by sonication over ice (three cycles of 30-s pulse at 20% intensity / 1 min pause). The solution was transferred to centrifuge bottles and centrifuged for at 18000 × g for 30 min at 4 ºC. During centrifugation, 1 mL of Ni-NTA resin was equilibrated in 40 mM phosphate buffer pH 8.0, with 300 mM NaCl. After centrifugation of the lysate, the supernatant was transferred to a column of Ni-NTA resin and incubated for 1 h while gently rotating at 4 ºC. After incubation, the flow through was discarded, followed by two column-volume washes and elution of bTG using 40 mM phosphate buffer pH 8.0, with 300 mM NaCl and 250 mM imidazole. Eluted bTG was dialyzed against 50 mM phosphate buffer pH 8.0. The average yield was 7 mg of activated bTG per litre of culture, with greater than 70% purity as estimated through evaluation of 10% SDS-PAGE (Figure 4.23) followed by staining with Coomassie blue. Aliquots were snap-frozen and stored at -80°C in 15% glycerol.

# Cloning and expression of mRuby2 and Clover into pACYCduet-1

Plasmids pCDNA3.1-mRuby2 and pCDNA3.1-Clover, coding for mRuby2 and Clover respectively, were purchased from Addgene. To maximize expression levels, both FPs were fused to maltose binding protein (MBP) to improve solubility and expression levels.

**Table B. Oligonucleotides used for cloning of Clover-6K**

| pCDNA3_Clover_Fw_NdeI | 5’ - AGT GTG CTG GAA TTC CAT ATG GTG - 3’ |
| --- | --- |
| pCDNA3_Clover_Rv_NdeI | 5’ - TAT AGA CAT ATG ATA GGG CCC TCT AGA CTT- 3’ |
| pMAL_Fw_ MfeI | 5’ - CGA GCA ATT GAC CAA CAA GGA CCA TAG ATT ATG - 3’ |
| pMAL_Clover_  spacer_Rv_NdeI | 5’ - TAT AGA CAT ATG GGA GGA GCC GGA GGA GCC GGG CCC TCT AGA - 3’ |
| pMAL_Clover_  spacer_6K_Rv_  NdeI | 5’ - TAT AGA CAT ATG TTT TTT TTT TTT TTT TTT GGA GGA GCC GGA - 3’ |
| pACYC_MBP_  Clover_Fw_Bg1II | 5’ – AAA AGA TCT C ATG AAA ATC GAA GAA - 3’ |
| pACYC_MBP_  Clover6K_Rv_EcoRV | 5’ - TAT AGA GAT ATC TTT TTT TTT TTT - 3’ |

**Table C. Oligonucleotides used for cloning of mRuby2-7M48**

| pCDNA3_mRuby2_Fw_NdeI | 5’ - GCC GCC ACC CAT ATG GTG CGG GGT- 3’ |
| --- | --- |
| pCDNA3_mRuby2_Rv_NcoI | 5’ – TTT CCA TGG GGC ACA GTC GAG GCT GAT - 3’ |
| pMAL_Fw_ MfeI | 5’ - CGA GCA ATT GAC CAA CAA GGA CCA TAG ATT ATG - 3’ |
| pMAL_mRuby2_  spacer_Rv_NcoI | 5’ – AGA CCA TGG GGA GGA GCC GGA GGA GCC GGC ACA GTC GAG- 3’ |
| pMAL_mRuby2_  spacer_7M48_Rv_NcoI | 5’ –TAT AGA CCA TGG ATG TGG ACG TTG TAA TGC CCA GGA GGA GCC GGA- 3’ |
| pACYC_MBP_  mRuby2_Fw_BamHI | 5’ - AGA TCT C G GGA TCC ATG AAA ATC GAA GAA - 3’ |
| pACYC_MBP_  mRuby2_7M48_Rv_EcoRI | 5’ –TAT AGA GAA TTC ATG TGG ACG TTG TAA TGC - 3’ |

**Table D. Oligonucleotides used for cloning of mRuby2-NMT**

| pCDNA3_mRuby2_Fw_NdeI | 5’ - GCC GCC ACC CAT ATG GTG CGG GGT- 3’ |
| --- | --- |
| pCDNA3_mRuby2_Rv_NcoI | 5’ – TTT CCA TGG GGC ACA GTC GAG GCT GAT - 3’ |
| pMAL_Fw_ MfeI | 5’ - CGA GCA ATT GAC CAA CAA GGA CCA TAG ATT ATG - 3’ |
| pMAL_mRuby2_  spacer_Rv_NcoI | 5’ – AGA CCA TGG GGA GGA GCC GGA GGA GCC GGC ACA GTC GAG- 3’ |
| pACYC_MBP_  mRuby2_Fw_BamHI | 5’ - AGA TCT C G GGA TCC ATG AAA ATC GAA GAA - 3’ |
| pACYC_MBP_  mRuby2_spacer_Rv_EcoRI | 5’ –TAT AGA GAA TTC GGA GGA GCC GGA GGA GCC - 3’ |
| pACYC_MBP_  mRuby2_spacer_NMT_Rv_EcoRI | 5’- TAT AGA GAA TTC **NMT NMT NMT** CTG **NMT NMT NMT** GGA GGA GCC GGA GGA - 3’ |

-**NMT** represents degenerate codon, where N=all four base pairs, M= adenine and cytosine, T=thymine

Forward and reverse primers were synthesized by IDT. Primers were designed to sub-clone the FP gene from pCDNA3.1 to the MCS of pMAL-c5X. FPs were sub-cloned out of pCDNA 3.1 template using PCR and ligated into pMAL-c5X MCS to create MBP-FP fusion protein. After sequencing confirmation by Eurofins Scientific, a GSSGSS spacer was introduced to the C-terminal of each FP using similar cloning techniques. Following sequencing of the MBP-FP-GSSGSS fusion protein, the substrate tag sequence (7M48, 6K or the NMT library) was introduced to the C-terminus of each construct. After successful assembly of the resulting MBP-FP-tag (tag= 7M48, 6K or the NMT library) construct, each variant was successively PCR amplified out of the pMAL-c5X, digested and ligated into the pACYC-duet1 vector using the appropriate restriction sites.

For the NMT library, the degenerate codon primers were used to code for peptide substrate sequence on the terminus of the FP. Due to the length of the reverse primers, touchdown PCR was necessary to amplify the gene of interest. After amplification of the MBP-mRuby2-GSSGSS-NMT gene, the gene was digested with BamHI and EcoRI and ligated in the pMAL-c5X vector. The ligated product was then transformed into chemically competent *E. coli*-bTG cells, which were then used to inoculate a 10-mL LB pre-culture.
